# On the Verge of a Catastrophic Collapse? The Need for a Multi-Ecosystem Approach to Microbiome Studies

**DOI:** 10.3389/fmicb.2021.784797

**Published:** 2021-12-02

**Authors:** Olaf F. A. Larsen, Linda H. M. van de Burgwal

**Affiliations:** Athena Institute for Research on Innovation and Communication in Health and Life Sciences, Vrije Universiteit Amsterdam, Amsterdam, Netherlands

**Keywords:** transitions, catastrophic collapse, planetary health, keystone taxa, microbial guilds

## Abstract

While the COVID-19 pandemic has led to increased focus on pathogenic microbes that cross the animal-human species barrier, calls to include non-pathogenic interactions in our perspective on public health are gaining traction in the academic community. Over generations, the diversity of the human gut microbiota is being challenged by external perturbations and reduced acquisition of symbiotic species throughout life. When such reduced diversity concerns not only the microbial species, but also the higher taxonomic levels and even the guild level, adequate compensation for possible losses may be lacking. Shifts from a high-abundance to a low-abundance state, known as a tipping point, may result in simultaneous shifts in covarying taxa and ultimately to a catastrophic collapse in which the ecosystem abruptly and possibly irreversibly shifts to an alternative state. Here, we propose that co-occurrence patterns within and between microbial communities across human, animal, soil, water, and other environmental domains should be studied in light of such critical transitions. Improved mechanistic understanding of factors that shape structure and function is needed to understand whether interventions can sustainably remodel disease-prone microbiota compositions to robust and resilient healthy microbiota. Prerequisites for a rational approach are a better understanding of the microbial interaction network, both within and inter-domain, as well as the identification of early warning signs for a catastrophic collapse, warranting a timely response for intervention. We should not forget that mutualism and pathogenicity are two sides of the same coin. Building upon the planetary health concept, we argue that microbiome research should include system level approaches to conserve ecosystem resilience.

HIGHLIGHTS

1. Non-pathogenic interactions between ecosystems play a key role in maintaining health.

2. The human gut microbiome may be on the verge of a catastrophic collapse.

3. Research should identify keystone taxa and guilds that interconnect different domains.

4. We should not forget that mutualism and pathogenicity are two sides of the same coin.

## Introduction

Host-microbe interactions have primarily been studied from a perspective of pathogenicity, and in light of COVID-19, attention has focused on a range of pathogenic microbes that cross the animal-human species barrier. Indeed, zoonotic epidemics urge us to critically reconsider our thinking on how to safeguard public health. Calls to include non-pathogenic interactions between microbes and hosts in our perspective on public health are gaining traction in the academic community (Cepon-Robins and Gildner, 2020). Here, we argue that this perspective should not only encompass an understanding of the microorganisms comprising the human ecosystem, but also those of other “non-human” ecosystems like animal, plants, water, and soil. As social norms influence innovation processes from both a push (e.g., what gets researched and developed) and pull (e.g., what is accepted by societal stakeholders) perspective, a thorough and early understanding of the societal impact of knowledge is important to guide and facilitate such processes (Van de Burgwal et al., 2018a; Hernando-Amado et al., 2020). This enables stakeholders to address unmet societal needs (Reperant et al., 2014; Van de Burgwal et al., 2016), to share and cooperate across the research and development process (Ribeiro et al., 2018; van der Waal et al., 2020), and to use best practices to prevent step-limiting roadblocks (van de Burgwal et al., 2018b; Flach et al., 2019).

## The Role of the Microbiome in Health Homeostasis

The human microbiota is increasingly acknowledged as one of the key players responsible for a robust and resilient healthy homeostasis, with “old friends” playing an essential role in the development and regulation of the mammalian immune system, host metabolism, and brain function (Ball et al., 2000; McDade et al., 2012; Rook et al., 2014; von Mutius, 2016; Hill and Round, 2021). With early life being commonly understood as a critical window for establishing a healthy microbiome (Cox et al., 2014), the acquisition and maintenance of essential microbial communities continue during adolescence and into adulthood (Riedler et al., 2001; Ege et al., 2008; Milo and Kahana, 2010; Rehman et al., 2010; Claesson et al., 2012). Indeed, the maintenance of a healthy gastrointestinal microbiome is considered critically dependent on the continuous acquisition of microorganisms and appropriate supporting substrates through food, water, air and other interactions with the environment (Dethlefsen et al., 2007; David et al., 2014).

The health of the human microbiota, however, is challenged through different mechanisms, with changes in microbial communities occurring in timescales as small as daily and as large as evolutionary (Banatvala et al., 1993; Hehemann et al., 2010; Yatsunenko et al., 2012). First, a decline or loss of certain species can be instigated as a result of external perturbations, such as antibiotic treatment and maladapted diets (David et al., 2014; Thorburn et al., 2014; Johnson et al., 2019). Second, improved hygiene has made it less likely that deficits are compensated through horizontal transmission, for example, fecal contamination of drinking waters or physical contact due to social crowding (Bach, 2021). Such lack of microbe replenishment has been correlated with frailty, upregulation of inflammatory markers, and reduced immune function (Claesson et al., 2012; Voorhies and Lorenzi, 2016). And third, opportunities for infants to acquire a healthy microbiome through vertical, that is, maternal transmission, are reduced as a result of these external perturbations and reduced horizontal transmission, compounding the effect (Blaser and Falkow, 2009; Pärnänen et al., 2018).

## Resilience Mediated By Keystone Taxa and Microbial Guilds

While a resilient microbiome recovers from such temporary changes to microbial diversity, persistent changes of microbiome composition lead to progressive loss of microbiome diversity over generations (Dethlefsen and Relman, 2011; Lichtman et al., 2016; Sonnenburg et al., 2016). Throughout history, we see a number of transitions occurring, with human gut microbiomes loosing key strains, and becoming less diverse. Generally, Western populations are characterized by an overall reduced microbial diversity and stability in their gut microbiome, as compared to unindustrialized rural communities (De Filippo et al., 2010; Yatsunenko et al., 2012), with human gut microbiomes from non-industrialized societies closer resembling ancient feces microbiota (Olm and Sonnenburg, 2021). These differences translate to reduced functionality, with, for example, less diverse repertoires for utilizing carbohydrates (Smits et al., 2017). It is also proposed that such changes are a key driver for the rise in inflammatory diseases throughout the 20th and 21st century (Bach, 2002, 2018) and other complex multifactorial diseases (Virgin and Todd, 2011; Huttenhower et al., 2012; Lemon et al., 2012; Faith et al., 2013; Kreiner et al., 2017; Kolodziejczyk et al., 2019; von Mutius and Smits, 2020). While functional redundancy between species in the human gut microbiota can serve as an “insurance” against decreases in abundance or functionality of certain species (Elmqvist et al., 2003; Nicholson et al., 2012; Shafquat et al., 2014; Sommer et al., 2017), reduced diversity ultimately leads to reduced resilience of the gut microbiota.

On a mechanistic level, we have come to understand that this functional diversity is not only mediated by certain species, but by keystone taxa and microbial guilds. Keystone taxa are defined as “*highly connected taxa that individually or in a guild exert a considerable influence on microbiome structure and functioning irrespective of their abundance across space and time. These taxa have a unique and crucial role in microbial communities, and their removal can cause a dramatic shift in microbiome structure and functioning*.” (Banerjee et al., 2018). The importance of keystone taxa is exemplified by the restoration of microbiome diversity that can be accomplished generations later by co-introduction of such keystone microbiota taxa with relevant dietary fibers (Sonnenburg et al., 2016). Microbial guilds, which microbial members may originate from different taxonomic backgrounds, are small ecosystems in which the individual taxa work together as coherent functional units or by exploiting the same class of resources (Wu et al., 2021). As guilds function as a single biological entity, the guilds in itself are expected to be particularly resilient. At the same time, simulations on hypothetical microbial guilds suggest that a decreased diversity within the guild leads to diminished efficiency of the physiological process the guild is dedicated to, as well as less stability (Larsen and Claassen, 2018). Using the microbial guild concept, it appears natural to extend the paradigm of functional diversity being related to the diversity of taxa further to the diversity of functional microbial guilds. As such, a higher functional diversity should be attributed to a higher diversity of functional microbial guilds, each guild dedicated to its own unique physiological process and hence its own functionality. Maintaining appropriate keystone taxa and “keystone guilds” can then be seen as necessary in order to safeguard both sufficient functional diversity as well as redundancy in functionalities enabling adequate compensation for possible losses in functional taxa and guilds.

## Tipping Points and Catastrophic Collapse

Whereas most taxa in the human gut microbiome are characterized by gradual variation, a number of specific taxa are characterized by the existence of bistable states of low or high abundance (Lahti et al., 2014). These taxa can undergo an abrupt shift from a high-abundance to a low-abundance state. Interestingly, shifts across tipping points of one taxa are often associated with similar shifts in covarying taxa (Lahti et al., 2014). Placing this notion of keystone taxa and “keystone guilds” in an evolutionary perspective, the successive extinction (and acquisition) of functionalities (traits) may cause a “catastrophic collapse,” leading to “abrupt and possibly irreversible shifts between alternative ecosystem states” (Dakos et al., 2019). Such large-scale collapses are characterized by “tipping points,” across which the ecosystems tips toward an alternative state. Not unlike the guild concept, these taxa may represent “tipping elements,” specific components that exhibit bistable states and are related to the overall health of the ecosystem (Lenton et al., 2008; Lahti et al., 2014).

Shifts across tipping points may be a consequence of disruptive selection, in which more extreme phenotypes have an advantage over intermediate phenotypes, be induced by environmentally or genetically determined host factors, or can occur as the result of ecological interactions between microbial communities (Rueffler et al., 2006; Faust and Raes, 2012). A proper assessment of current health problems like new pandemics and the rise of autoimmune indications can thus only be effective by incorporation of all (microbial) ecosystems involved, not being limited to our human microbiome alone. For example, a full comprehension of zoonotic disease requires not only understanding of the human microbiome but also of the microbial ecosystem belonging to the vector from which the pathogenic microorganism is being transferred, as also suggested by the concept of microbiota coalescence (Baquero et al., 2021). Analogously, health cannot be understood without a proper understanding of the ecosystem containing the old friends. For example, it was recently suggested that coinfection with soil-transmitted helminths may suppress the inflammatory response upon infection with SARS-CoV-2 (Cepon-Robins and Gildner, 2020). Hence, a full comprehension of our homeostasis (and threats to it) can only be reached through a broader perspective, in which the bidirectional interactions between the mutualistic microbial ecosystems involved (human gut, skin, animal, soil etc.) are being understood.

## A Wider Perspective: Co-Occurrence Patterns in a Planetary Health Context

The planetary health concept naturally urges for a wider view with respect to cherishing our old friends, which is indeed not limited to the human gut microbiota anymore. Just as microbiome communities within humans are characterized by their robustness and resilience, so are the broader ecosystems in which we live (McFall-Ngai et al., 2013). Paradoxically, while the widespread use of antibiotics has led to the propagation of antimicrobial resistance genes as well as increased density of mobile genetic elements, implying accelerated evolutionary rates (Baquero, 2021), changes in our way of living have led to reduced replenishments of symbiotic bacteria. Indeed, not only contact with microbiota from other humans and animals are of relevance, so are material and urban microbial populations, which are indicated to play a role in, for example, “sick building syndrome” (Sahlberg et al., 2010) and antimicrobial resistance (Danko et al., 2021). The planetary health concept underscores the close relation between human health within a flourishing natural system, propagating across-ecosystem approaches to understand the links between health and environmental change (Whitmee et al., 2015).

The possibility of a future catastrophic collapse, through the role of the environmental microbiome in the development and propagation of antibiotic resistance genes, is now broadly recognized (Robinson et al., 2016; Jørgensen et al., 2018; Thakur and Gray, 2019). Attention has been given to less biodiverse human environments, and a need to “Rewild” urban green spaces, allowing interactions with beneficial microbiome in the direct environment of humans (Mills et al., 2017). Green space exposure has been shown to increase diversity in skin and nasal microbiota (Selway et al., 2020). Likewise, living in close proximity to natural environments has been linked to mental health – a relation that may, at least in part, be mediated by contact with environmental microbiota (Flandroy et al., 2018).

Co-occurrence patterns within and between microbial communities, however, have to-date been relatively sparsely studied (Williams et al., 2014; Klimenko et al., 2020). In addition, the research on critical transitions in the gut microbiome is still in its early stages and primarily related to the gut microbiome within individuals (Tytgat et al., 2019; Malard et al., 2020). Moreover, modeling microbial communities are still faced with numerous challenges (Faust and Raes, 2012; Gil-Gil et al., 2021). A step beyond, manipulating and engineering microbial communities, a process described as synthetic ecology, is even more early stage (Dunham, 2007; Zomorrodi and Segrè, 2016). Whereas some research has studied the role of keystone taxa in critical transitions in water microbiomes (Wang et al., 2020), their role is not yet described in critical transitions within host microbiomes nor in gut microbiomes across the human species.

## Discussion

Following all this, we advocate the investment in strong research efforts toward the identification of keystone taxa and (keystone) microbial guilds that interconnect the different domains (ranging from human, animal, soil, water, and other environmental domains), altogether responsible for a balanced ecosystem that is both robust toward potential communicable and non-communicable disease, as to the minimization of pathogenic transmissions between these domains. A key question in microbiota research is whether such interventions can sustainably remodel disease-prone microbiota compositions to robust and resilient healthy microbiota compositions (Sanders et al., 2013). Probiotic microorganisms can then be regarded as “supplemental keystone taxa.” Improved mechanistic understanding of factors that shape structure and function is needed to further design interventions with pre- and probiotics (Bäckhed et al., 2012).

A first prerequisite for such a rational approach toward intervention is a better understanding of the microbial interaction network, both within species and between species. The subdivision of the interactions between mutualistic ecosystems into different subsets each characterized by a topological parameter like the *k*-core [a *k*-core indicates that all nodes (taxa) of the associated subgraph have a direct connection to at least *k* nodes] is demonstrated to be a highly useful parameter. In particular, taxa belonging to the maximum *k*-core (highest connectivity) are crucial for the ecosystems stability, and removal of these taxa results in a total collapse of the complete network (Morone et al., 2019). We propose that the same may account for microbial guilds: keystone guilds will have highest connectivity to other guilds and should also be cherished to prevent possible catastrophic collapses. In addition, replenishment of additional species may be supportive to the formation and maintenance of these keystone guilds.

A second prerequisite is the identification of early warning signs for a catastrophic collapse, warranting a timely response for intervention. Such early warning signs have been described in detail (Scheffer et al., 2009), and include a slowing down of the system (exemplified by, a slower recovery of the ecosystem to its original state after an external perturbation, increased autocorrelation, and increased variance in the pattern of fluctuations of the ecosystem). All the aforementioned should be taken into perspective when monitoring changes in gut health. As such, the identification of these topological parameters and warning signs will enable us to selectively target the restoration and reinforcement of these entities in order to prevent an outbalanced ecosystem leading to a possible overall catastrophic collapse.

## Conclusion

**Figure 1:**
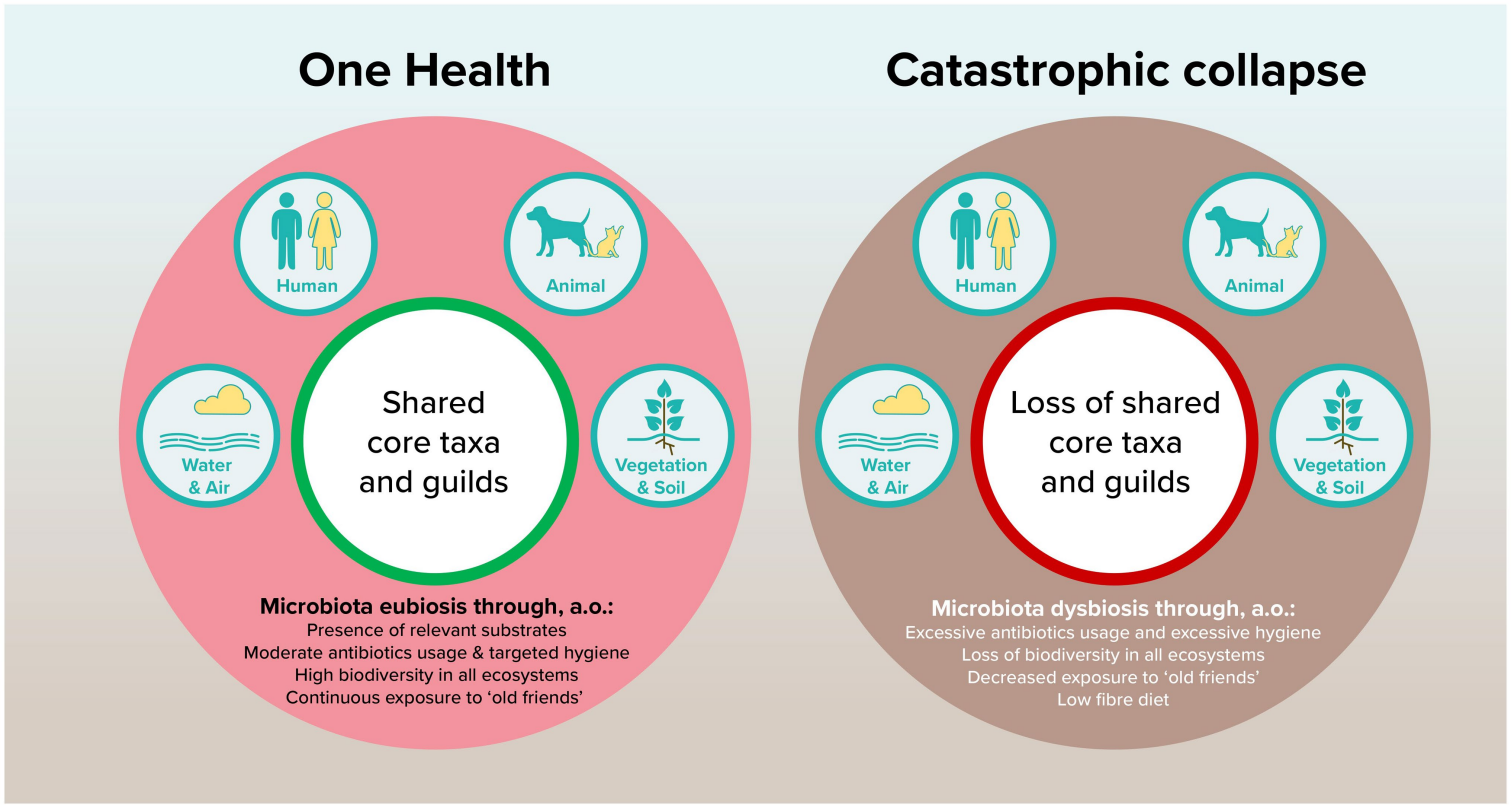
Proposed model of how shared core taxa and guilds can contribute to planetary health through microbiota eubiosis.

We should not forget that mutualism and pathogenicity are two sides of the same coin, see Figure 1. Whereas currently much attention has been given to the risk of zoonotic pathogens, we must be careful to not isolate ourselves from our microbial environment. The evolution in the human microbiome that is visible over decades and across populations is a starting point to investigate the resilience and robustness of healthy human gut microbiomes. Without careful consideration, we may be on the verge of a catastrophic collapse on a population level, leading to functional decline of gut ecosystems. Building upon the planetary health concept, we argue that research on the microbiome should not only focus on the individual level through personalized approaches, but even more on the system level to conserve ecosystem resilience.

## Data Availability Statement

The original contributions presented in the study are included in the article/supplementary material, and further inquiries can be directed to the corresponding author.

## Author Contributions

OL: conceptualization, formal analysis, and writing – review and editing. LB: conceptualization and writing – original draft. All authors contributed to the article and approved the submitted version.

## Conflict of Interest

OL is also Senior Manager Science at Yakult. LB is consultant for several commercial parties in the field of probiotics and life sciences; none of her advising practices are related to or in conflict with the content of this research.

## Publisher’s Note

All claims expressed in this article are solely those of the authors and do not necessarily represent those of their affiliated organizations, or those of the publisher, the editors and the reviewers. Any product that may be evaluated in this article, or claim that may be made by its manufacturer, is not guaranteed or endorsed by the publisher.
